# Variation in herpetofauna detection probabilities: implications for study design

**DOI:** 10.1007/s10661-021-09424-0

**Published:** 2021-09-17

**Authors:** Jeremy A. Baumgardt, Michael L. Morrison, Leonard A. Brennan, Madeleine Thornley, Tyler A. Campbell

**Affiliations:** 1 Texas A&M Natural Resources Institute, 578 John Kimbrough Boulevard, College Station, TX 77843 USA; 2 Department of Rangeland, Wildlife and Fisheries Management, Texas A&M University, College Station, TX 2258 TAMU77843 USA; 3 Caesar Kleberg Wildlife Research Institute, Texas A&M University-Kingsville, MSC 218, 700 University Boulevard, Kingsville, TX 78363 USA; 4 Department of Rangeland, Wildlife and Fisheries Management, Texas A&M University, College Station, TX 77843 USA; 5 East Foundation, 200 Concord Plaza Drive, Suite 410, San Antonio, TX 78216 USA

**Keywords:** Amphibians, Capture rates, Monitoring, Occupancy modeling, Reptiles

## Abstract

Population monitoring is fundamental for informing management decisions aimed at reducing the rapid rate of global biodiversity decline. Herpetofauna are experiencing declines worldwide and include species that are challenging to monitor. Raw counts and associated metrics such as richness indices are common for monitoring populations of herpetofauna; however, these methods are susceptible to bias as they fail to account for varying detection probabilities. Our goal was to develop a program for efficiently monitoring herpetofauna in southern Texas. Our objectives were to (1) estimate detection probabilities in an occupancy modeling framework using trap arrays for a diverse group of herpetofauna and (2) to evaluate the relative effectiveness of funnel traps, pitfall traps, and cover boards. We collected data with 36 arrays at 2 study sites in 2015 and 2016, for 2105 array-days resulting in 4839 detections of 51 species. We modeled occupancy for 21 species and found support for the hypothesis that detection probability varied over our sampling duration for 10 species and with rainfall for 10 species. For herpetofauna in our study, we found 14 and 12 species were most efficiently captured with funnel traps and pitfall traps, respectively, and no species were most efficiently captured with cover boards. Our results show that using methods that do not account for variations in detection probability are highly subject to bias unless the likelihood of false absences is minimized with exceptionally long capture durations. For monitoring herpetofauna in southern Texas, we recommend using arrays with funnel and pitfall traps and an analytical method such as occupancy modeling that accounts for variation in detection.

## Introduction

Global biodiversity is being lost at a rapid rate (Pimm et al., 2014). Monitoring populations of wildlife is fundamental for conservation and is necessary for evaluating the effectiveness of past management strategies and to inform future management decisions. Due to the paucity of resources available for conservation efforts, identifying and employing efficient monitoring methods are often a primary objective. To be useful, however, a monitoring program needs to be capable of detecting a change in population abundance early enough to allow for adjustments in management actions to reach conservation goals. Thus, the efficiency of a monitoring strategy must be balanced with the ability to meet specified objectives. Failure to design monitoring capable of meeting objectives will result in wasting limited resources (Legg & Nagy, 2006; Williams et al., 2002).

Species richness has been used to identify areas of high conservation value (Meyers et al., 2000) and thus has value as a monitoring metric. Furthermore, methods to efficiently estimate and monitor species richness have been used for groups such as tropical birds (Herzog et al., 2002; MacLeod et al., 2011) and arthropods (Orbist & Duelli, 2010). While species richness is commonly used to measure and detect changes in biodiversity, evidence suggests that species richness estimates can be uninformative and, perhaps, misleading. The dependency of species richness measures on both spatial and temporal scales is often overlooked; thus, comparisons over space and time may be invalid (Fleishman et al., 2006; Hillebrand et al., 2018). Furthermore, reliance on data based on raw species lists for monitoring or estimating species richness is highly susceptible to bias from variations in detection probability and may lead to erroneous conclusions (Boulinier et al., 1998; Fleishman et al., 2006). Numerous unbiased methods have been developed that attempt to estimate richness by accounting for species not detected, such as the jackknive estimator, the capture-recapture framework, and species accumulation and rarefaction curves (Boulinier et al., 1998; Colwell & Coddington, 1994; Dorazio et al., 2006; Gotelli & Colwell, 2001; Heltshe & Forrester, 1983). However, these methods may be unsatisfactory as they do not identify the species that were not detected, but hypothetically present. Thus, these methods have limited utility for addressing questions regarding community dynamics that require knowledge of the true community composition.

Amphibians and reptiles (hereafter, herpetofauna) have been experiencing declines worldwide and suffer from a chronic lack of data (Alroy, 2015; Böhm et al., 2013). Monitoring herpetofauna is difficult, requiring sampling methodologies that cater to a diversity of species that may be fossorial, cryptic, or seasonally dormant. As a result, much effort has been focused on comparing efficacy of various capture and detection methods with an emphasis on methods that are effective for a large number of species (e.g., Hutchens & DePerno, 2009; Michael et al., 2012; Ryan et al., 2002). However, recommendations on the best metric for monitoring herpetofauna communities remain unclear. Some monitoring programs for herpetofauna appear to focus on simple counts without further calculations of detection probabilities (Heyer et al., 1994; Smith & Petranka, 2000; JNCC, 2004; Hare, 2012). Yet others emphasize the need for robust methods that generally require more effort (Dodd & Dorazio, 2004; Hyde & Simons, 2001; Muths et al., 2006). This additional effort may be cost-prohibitive; thus, identification of methods that promote estimation of robust metrics and efficiently provide adequate sample sizes for multiple species is essential.

The South Texas Plains is a unique brush country ecosystem containing semi tropical, grassland, and desert species of herpetofauna. This thorn scrub region is home to species of concern, including Texas tortoise (*Gopherus berlandieri*), Texas horned lizard (*Phrynosoma cornutum*), and Texas indigo snake (*Drymarchon melanurus erebennus*). Our objectives were to estimate species-specific detection probabilities using an occupancy modeling framework with a common and easily reproducible trap array for a large and diverse group of amphibians and reptiles. The primary motivation for this was to provide the information for developing an efficient monitoring program capable of detecting population changes early enough to allow for adjustments in management actions to reach conservation goals. Our secondary objective was to evaluate the relative effectiveness for South Texas of 3 common trapping devices that are often incorporated into a single trap array; funnel traps, pitfall traps, and cover boards.

## Materials and methods

### Study area

**Fig. 1:**
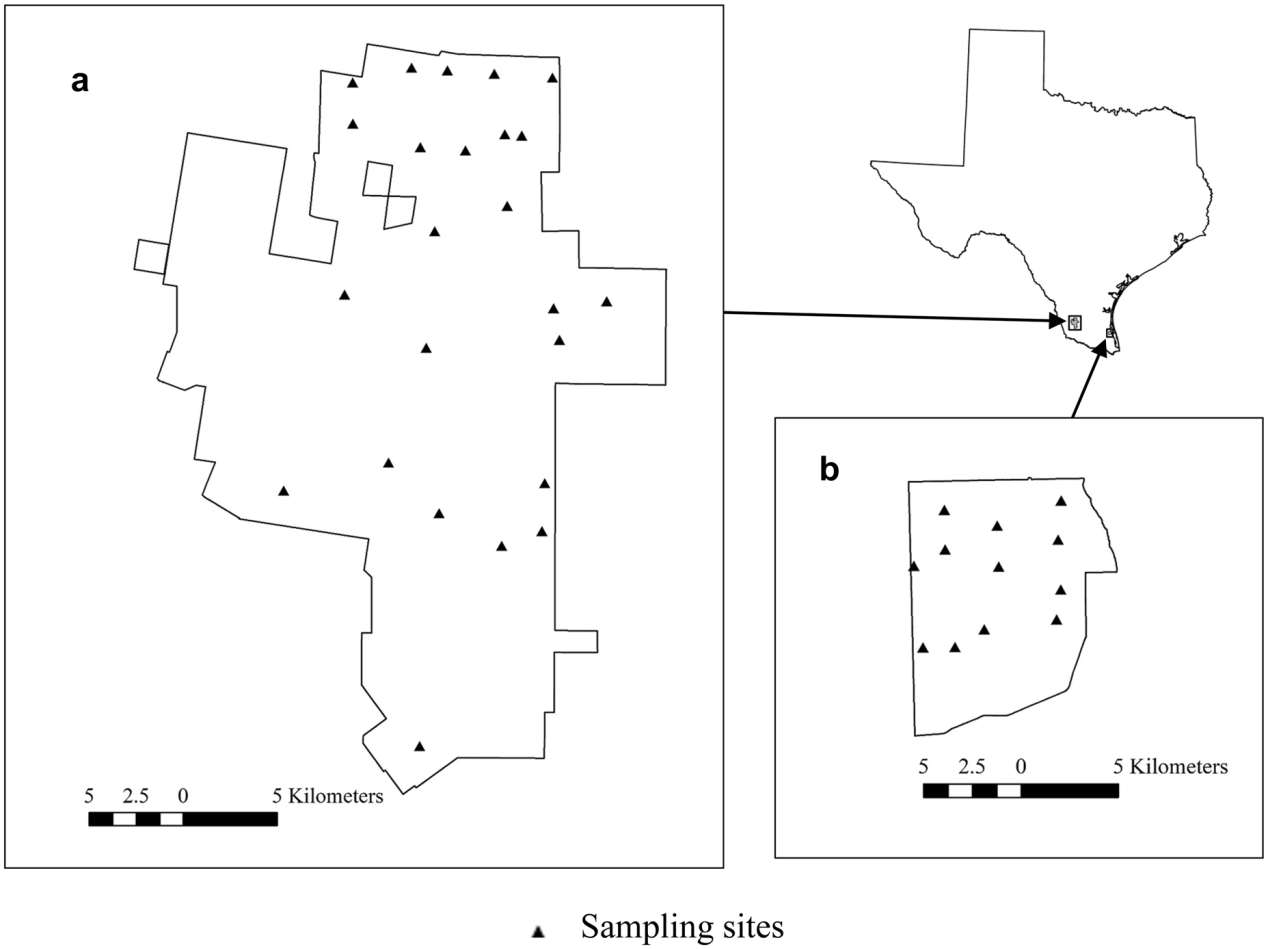
Location of sampling areas on the East Foundation’s San Antonio Viejo Ranch (**a**) and the El Sauz Ranch (**b**), TX, USA, 2015–2016

We conducted our research on 2 study sites in southern Texas, USA; the San Antonio Viejo Ranch (hereafter SAV) located in Jim Hogg and Starr counties and the El Sauz Ranch (hereafter ELS) located in Willacy and Kenedy counties (Fig. 1). Both sites are owned by the East Foundation, which is an agricultural research organization that promotes land stewardship and manages a cow-calf operation for research and education (Montalvo et al., 2020). The SAV was approximately 61,000 ha of a matrix of woodland (73%), shrubland (18%), and grassland (5%). The ELS was approximately 11,000 ha adjacent to the Laguna Madre along the Texas Gulf Coast and was a matrix of woodland (36%), wetland vegetation (30%), grassland (27%), and shrubland (5%). Annual rainfall was highly variable in the region; mean annual rainfall on was 57 cm for SAV with a mean daily high temperature of 29 °C (NOAA, 2016). The ELS site was slightly wetter and cooler with a mean annual rainfall of 66 cm and a mean daily high of 26.5 °C (NOAA, 2016).

### Data collection

We used common trap arrays consisting of pitfall traps, funnel traps, and cover boards with drift fencing (Jones, 1986). Each array consisted of three arms radiating 15 m from a central point. We placed pitfall traps made from 3.8-L (5 gallon) buckets at the center of the array and at the distal end of each arm. We buried each bucket such that the rim was at or slightly above grade to prevent them from filling with water during heavy rains. Additionally, we placed wire-mesh funnel traps on both sides of the drift fence, approximately 7.5 m from the central pitfall trap on all three arms. Funnel traps were constructed of vinyl-dipped steel mesh, 45-cm long with a diameter of 21.5 cm and an opening diameter of 3 cm. We placed funnel traps flush with the drift fence and pushed them into the sand until the distal rims were flush with the sand and no gap remained between the trap, drift fence, and ground. For our drift fencing, we used 91.4-cm (3 ft) silt fencing buried 10–15 cm in the ground and supported by wood stakes between the central and each of the 3 distal pitfall traps. We provided each pitfall and funnel trap with a plywood board for shade. We used substrate such as sticks and rocks to support shade boards 2–5 cm above the pitfall trap rims and leaned boards at a 45° angle against the drift fencing to shade the funnel traps. We also placed a 0.6 × 1.2 m sheet of plywood directly on the ground within 5 m of each of the three distal pitfall traps as cover boards as a third method of detection (Tietje & Vreeland, 1997).

We randomly located a single sampling site in each of 10 pastures associated with a long-term grazing study being conducted in the Coloraditas Grazing Research and Demonstration Area (Montalvo et al., 2020) on the northern 7500 ha of SAV (Fig. 1) and randomly located an additional 14 arrays over the remaining portion of SAV. Similarly, we randomly located 12 arrays across ELS for a total of 36. We restricted all locations to ≥ 150 m from external boundaries and ≥ 750 m from other sampling locations.

We trapped 18 April through 14 August in 2015 and 17 April through 6 August in 2016. We attempted to monitor 10–15 arrays simultaneously with staggered start dates such that no more than 3 arrays had the same start date. We attempted to monitor each array between 2 and 6 consecutive weeks with an average of 4 consecutive weeks. We closed an array by filling pitfall traps with soil and removing funnel traps if the area received heavy rains that resulted in standing water at the site or severely hindered access to the area. We reopened arrays as soon as the water receded and access was possible. We checked each array between 11:00 and 17:00 to minimize time animals spent in traps since we expected activity of most species to be greatest during the day. We released all captured animals within 1–2 m of their respective trap and directed them away from the trap array.

In a study in southern Texas, Ruthven et al. (2002) found amphibians were more active in summer, snakes were more active in spring, and lizards were equally active in spring, summer, and fall, with their greatest overall diversity detected in spring and summer. Since we were interested in detecting as many species of amphibians and reptiles as possible with our arrays, we intended to span the majority of the spring and summer seasons with our trapping effort.

### Data analysis

Occupancy (Psi) is defined as the proportion of sample sites occupied by a particular species. Naïve estimates of Psi are biased by assuming a species is absent if not detected, regardless of effort spent searching. Detection probability (*p*) is defined as the probability of detecting the species for a specified unit of effort, given the species occupies the area sampled. Occupancy modeling typically uses repeat surveys at the same location to produce an estimate of *p*, which is then used to generate an unbiased estimate of Psi (MacKenzie et al., 2006). We attempted to model occupancy for species with a minimum of 20 detections in at least 1 year of our study. We estimated Psi for each species for the 2 study sites and 2 years using the simple occupancy model in Program MARK (White & Burnham, 1999). Furthermore, we assumed *p* may vary for some species with time over our nearly 4-month field season (Ruthven et al., 2002; Todd et al., 2007) and may also vary due to rainfall (Jones, 1986). Thus, we considered linear and quadratic effects of ordinal date, as well as linear and quadratic effects of rainfall on *p*. We used daily precipitation totals recorded by the NOAA Global Historical Climatology Network for Escobas, TX (for SAV), and for Port Mansfield, TX (for ELS; NOAA, 2020), as covariates. We suspected the impact of rain on the activity of amphibians might have lasted > 1 day. Thus, we also considered linear and quadratic effects of accumulated rainfall for the 5 and 10 days leading up to the day of capture. We included a term for year and site, as well as the interaction for year and site for Psi in all our models such that resulting estimates for Psi could vary between years and sites for each species. We considered a species was observed at a site regardless of the style of trap it was captured in and we used each day of captures as a single observation, which resulted in estimates of *p* for a 24-h period for a single array.

We fit 18 models (Online Resource 1) to each species’ complete dataset that included all days of trapping for both years. We used Akaike’s information criterion adjusted for small sample sizes (AIC_c_; Burnham & Anderson, 2002) to identify the model with the most support for each species’ dataset, which we used to report estimated daily detection probabilities (*p*). The cumulative detection probabilities (p*) is defined as the probability of detecting the species at least once over a given period of sampling, given the species occupies the area (MacKenzie et al., 2006). We used the estimates for *p* to calculate p* for 4 and 10 days of trapping using the following equation.

$$p* =1-\left(\prod_{i=1}^{k}(1-{p}_{i})\right)$$

where *p*_*i*_ is the day-specific *p* and *k* = 4 or 10 days. Additionally, we calculated the number of days of trapping (*k*) that would be required to reach p* of 0.8 and 0.95. Finally, we predicted p* for hypothetical 10-day trapping periods over a range of dates and rainfall totals separately to show the potential impact these variables have on sampling success based on the models with the largest support for individual species.

We further evaluated the relative effectiveness of each of the 3 styles of traps we employed for each species we detected. We combined datasets for both study areas and both years and used a chi-square test of the hypothesis that captures were independent of trap type (Zar, 1984). For species with captures dependent on trap type, we calculated the relative risk (Agresti, 1996) by dividing the proportion of captures of a particular species in each of the trap types by the proportion of total traps made up by the type and reported resulting odds ratios to show the relative likelihood of capturing a species with each of the trap types.

## Results

We monitored each of the 36 arrays for a mean of 30 days with a minimum and maximum duration of 15 and 38 days, respectively, for a total of 1081 array-days in 2015. We removed one of the arrays on SAV from our study in 2016 due to difficult access. We monitored the remaining 35 arrays for a mean of 29.3 days with a minimum and maximum duration of 18 and 36 days respectively, for 1024 total array-days. Total precipitation during our sampling periods were 17.8 cm and 31.4 cm for ELS and SAV, respectively, in 2015, and 18.2 cm and 13.6 cm for ELS and SAV, respectively, in 2016.

We captured a total of 4839 individuals of 51 species over both years of the study, with 2337 and 2502 captures on ELS and SAV, respectively (Table 1). Our most commonly captured species on ELS were Rio Grande leopard frog (*Lithobates berlandieri*; 27%), Great Plains narrow-mouthed toad (*Gastrophryne olivacea*; 14%), and six-lined racerunner (*Aspidoscelis sexlineata*; 13%). Our most commonly captured species on SAV were six-lined racerunner (31%), eastern spotted whiptail (*Aspidoscelis gularis*; 24%), and keeled earless lizard (*Holbrookia propinqua*; 16%).

### Occupancy estimation

We sampled a total of 21 species (8 frogs, 7 lizards, and 6 snakes) with a minimum of 20 detections in at least 1 of our sampling years, which we used to generate occupancy and detection probability estimates (Table 2). Our sampling period for the ELS site was limited to a similar 6-week period (24 May–3 July) for both years of our study. Thus, for species we only detected on ELS (Mexican chirping frog, *Syrrhophus cystignathoides*; Rio Grande leopard frog; and sheep frog, *Hypopachus variolosus*), we did not attempt to estimate occupancy on SAV, nor did we attempt to predict *p* outside of this timeframe.

**Fig. 2:**
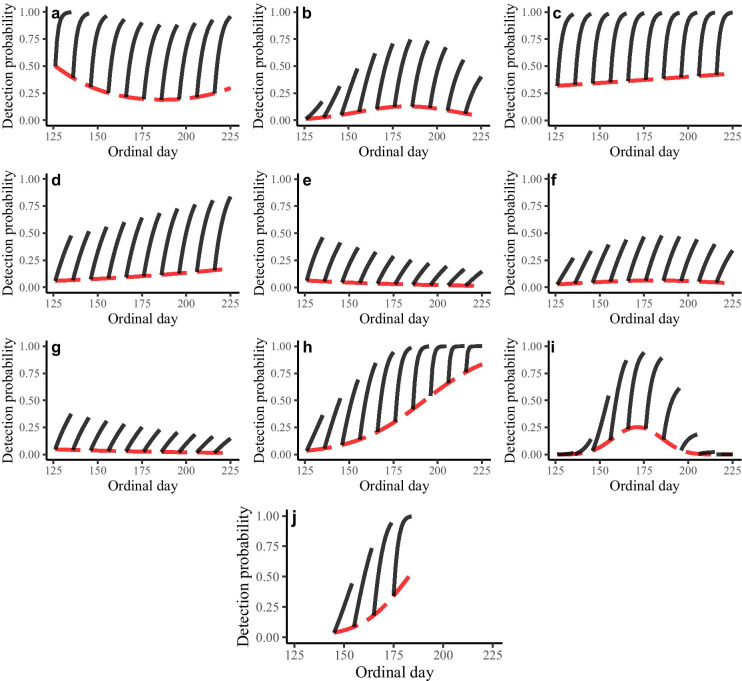
Capture probability estimates (red dashed line) and predicted cumulative detection probability for 10 hypothetical 10-day capture periods (black solid lines) from ordinal day 126 (5 May) through 226 (13 August) for species supporting an effect of date on detection probability from southern Texas, USA, 2015–2016. Species are **a** eastern spotted whiptail (*Aspidoscelis gularis*), **b** Great Plains skink (*Plestiodon obsoletus*), **c** six-lined racerunner (*Aspidoscelis sexlineata*), **d** Texas horned lizard (*Phrynosoma cornutum*), **e** flat-headed snake (*Tantilla gracilis*), **f** Texas glossy snake (*Arizona elegans arenicola*), **g** Texas nightsnake (*Hypsiglena jani texana*), **h** Great Plains narrow-mouthed toad (*Gastrophryne olivacea*), **i** Gulf Coast toad (*Incilius nebulifer*), and **j** Mexican chirping frog (*Syrrhophus cystignathoides*). Predictions were made using our average observed rainfall from both years (daily rainfall = 0.24 cm, 5-day rainfall accumulation = 0.98 cm, and 10-day rainfall accumulation = 1.87 cm) Note that we only detected Mexican chirping frogs on the El Sauz study site; thus, we restricted our predictions to the timeframe in which we sampled this area (ordinal day 145–184)

Of the 21 species we attempted to fit occupancy models to, 5 did not support any time- or rain-related covariates for describing variation in *p* (Table 2). A linear relationship with time was supported for 2 lizards, 2 snakes, and 2 frogs (Table 2, Fig. 2). Six-lined racerunner (Fig. 2c), Texas horned lizard (Fig. 2d), Great Plains narrow-mouthed toad (Fig. 2h), and Mexican chirping frog (Fig. 2j) all indicated an increasing *p* through our sampling timeframe, whereas flat-headed snake (*Tantilla gracilis*; Fig. 2e) and Texas night snake (*Hypsiglena jani texana*; Fig. 2g) both indicated a slight decline in *p* through time. Data for eastern spotted whiptail (Fig. 2a), Great Plains skink (*Plestiodon obsoletus*; Fig. 2b), Texas glossy snake (*Arizona elegans arenicola*; Fig. 2f), and Gulf Coast toad (*Incilius nebulifer*; Fig. 2i) all supported a quadratic relationship between *p* and time, with a peak in *p* ranging from our first date of capture (17 April) for eastern spotted whiptail to 2 July for Great Plains skink.

**Fig. 3:**
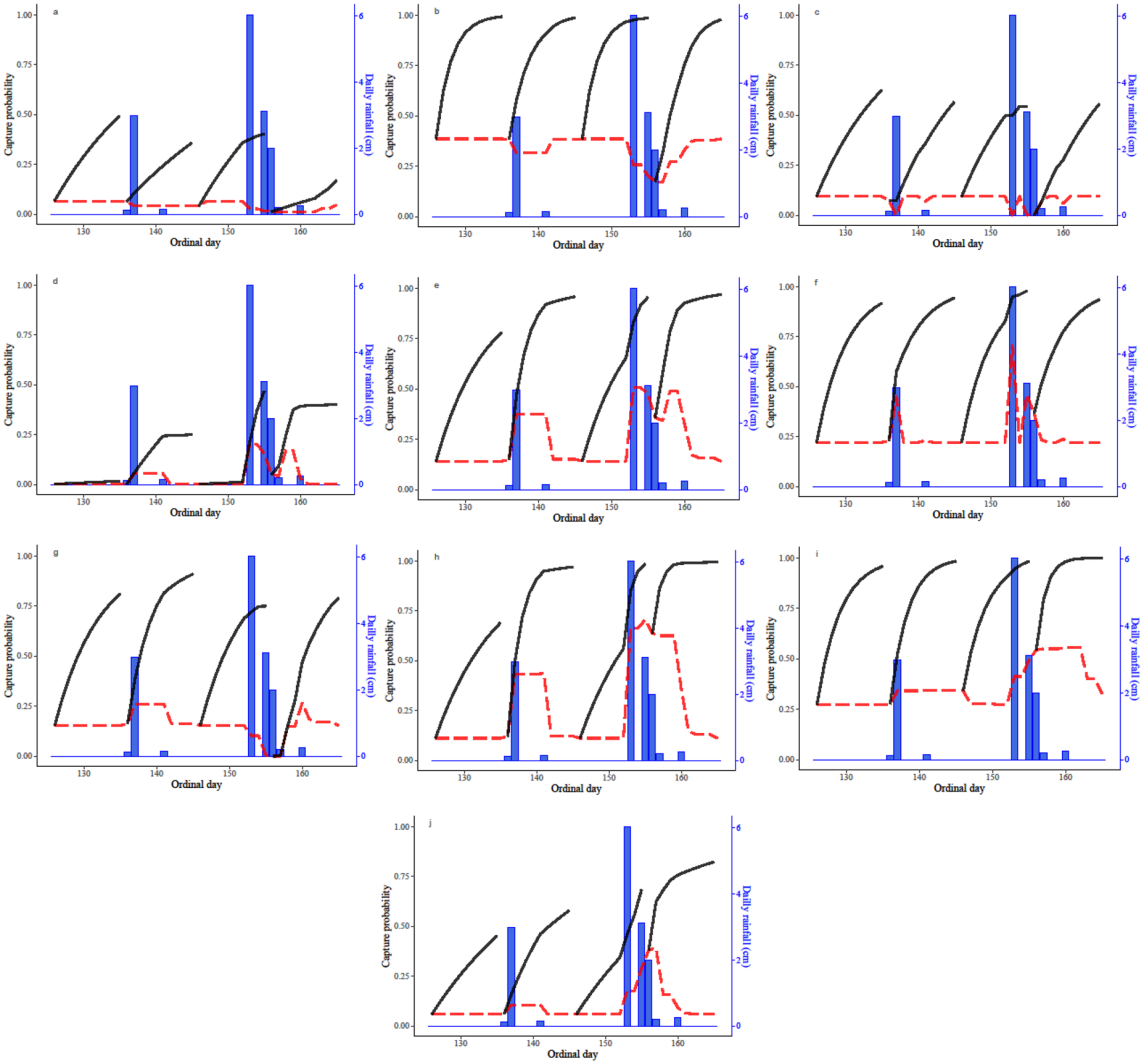
Capture probability estimates (red dashed line) and cumulative detection probability for 4 hypothetical 10-day capture periods (solid black lines; left axis) with our observed rainfall from Port Mansfield, TX, for the period 5 May 2016 through 13 June 2016 (blue bars; right axis) using the mean trap date of 15 June (ordinal day 166) for species supporting an effect of rain on detection probability from southern Texas, USA, 2015–2016. Species are **a** eastern fence lizard (*Sceloporus undulatus*), **b** six-lined racerunner (*Aspidoscelis sexlineata*), **c** Texas glossy snake (*Arizona elegans arenicola*), **d** Couch’s spadefoot (*Scaphiopus couchii*), **e** Great Plains narrow-mouthed toad (*Gastrophryne olivacea*), **f** Gulf Coast toad (*Incilius nebulifer*), **g** Hurter’s spadefoot (*Scaphiopus hurterii*), **h** Mexican chirping frog (*Syrrhophus cystignathoides*), **i** Rio Grande leopard frog (*Lithobates berlandieri*), and **j** sheep frog (*Hypopachus variolosus*)

We found support for a relationship between *p* and some measure of rainfall for 10 of the 21 species we attempted to fit models to (Table 2, Fig. 3). The covariate of daily rainfall was supported for Texas glossy snake (Fig. 3c) with a negative relationship, and for Gulf Coast toad (Fig. 3f) with a positive relationship. The 5-day accumulation of rainfall was supported by our data, either as a linear or quadratic relationship with *p*, for 1 lizard and 5 frogs (Table 1). The data for six-lined racerunner supported a linear and decreasing relationship between the 5-day total rainfall and *p* (Fig. 3b). Sheep frog, conversely, supported a positive linear relationship, with an estimated *p* near 0.10 with no rain in the 5-day period, increasing to a *p* around 0.40 with 12 cm of cumulative rainfall (Fig. 3j). Of the species supporting a quadratic relationship with the 5-day period of rain, a maximum *p* was predicted around 3 cm for Hurter’s spadefoot (*Scaphiopus hurterii*; Fig. 3g), 7 cm for both Great Plains narrow-mouthed toad (Fig. 3e) and Couch’s spadefoot (Fig. 3d), and 8 cm for Mexican chirping frog (Fig. 3h). Two species supported a model with a linear relationship between *p* and rain in the 10-day period leading up to the day of capture; eastern fence lizard (*Sceloporus undulates*) had a negative relationship (Fig. 3a), whereas Rio Grande leopard frog had a positive relationship (Fig. 3i).

### Cumulative detection probability

Our calculations of the cumulative detection probability (p*) for 4 days of sampling based on our model estimates for *p* ranged from 0.02 for Couch’s spadefoot (*Scaphiopus couchii*) to 0.84 for six-lined racerunner (Table 3). Our calculations for 10-day p* for these same species were 0.06 and 0.99, respectively. According to our estimates, it would be possible to reach a p* = 0.80 for 8 species if 8 days of sampling were used. Similarly, a p* = 0.95 could be reached for these same 8 species if the duration was increased to 15 days.

We plotted estimates of *p* and predicted p* for 10-day, hypothetical capture events over our range of capture dates of 5 May (ordinal date 126) through 13 August (ordinal date 226), while holding rain values at our observed means from both years (daily rainfall = 0.24 cm, 5-day rainfall = 0.98 cm, and 10-day rainfall = 1.87 cm) to show the impact of timing on predicted success. For species with evidence that *p* varied with time, but was relatively high across our sampling period (six-lined racerunner, Fig. 2c; eastern spotted whiptail, Fig. 2a), there was little impact on p* after a 10-day capture period. Alternatively, species with time effects and a relatively low *p* (< 0.15) over our capture period, had substantial variation in p* over a 10-day period. Our estimates of *p* for Great Plains skink ranged from 0.01 to 0.13 across our study period, resulting in 10-day p* estimates ranging from 0.17 to 0.74 (Fig. 2b). Our results for Gulf Coast toad suggest a small window of time when *p* reached a maximum of 0.25 around 19 June (ordinal date 171); trapping 10 days including this date would result in p* ~ 0.94, whereas trapping a month earlier would result in p* < 0.15 and a month later would result in p* < 0.20 (Fig. 2i).

We used the mean trap date of 15 June (ordinal date 166) and our observed rainfall from Port Mansfield, TX, for the period 5 May 2016 through 13 June 2016 to show the impact of rainfall on predicted p*. We selected this period because it began with 10 days without rain, followed by 30 days with a rainfall pattern representative of the location and season. Considering species with relatively high *p* (six-lined racerunner, Fig. 3b; Rio Grande leopard frog, Fig. 3i), there was little impact of rainfall on p* after a 10-day capture period, despite the variation in rainfall among periods. Similarly, species with a relationship with the single-day rainfall and a moderate *p* (Gulf Coast toad; Fig. 3f) and even a low *p* (Texas glossy snake; Fig. 3c) had little variation in predicted p* with rainfall patterns typical of our study area.

Alternatively, species with a moderate or low *p* and a relationship with the 5- or 10-day accumulation of rain typically showed increased variation in the predicted p*. Our predictions showed that capture periods with some rainfall would be substantially more efficient than without for Couch’s spadefoot (Fig. 3d), Mexican chirping frog (Fig. 3h), Great Plains narrow mouthed toad (Fig. 3e), and sheep frog (3j). Eastern fence lizard had a relatively low p and a negative relationship with the 10-day accumulation of rain, resulting in predicted p* ranging from 0.49 in the first period with no rain to 0.17 in the final period (Fig. 3a).

### Efficacy of trap types

We captured 61 individuals with cover boards, 2941 with funnel traps, and 1837 with pitfall traps (Table 1). Considering all captures without regard to species, the relative risk was 0.06, 1.32, and 1.23 for cover boards, funnel traps, and pitfall traps, respectively (Table 4). The observed frequencies for captures in each trap type were significantly different than would be expected if captures were independent of trap type for 31 species at the 0.05 level of significance. The relative risk for cover boards for all 31 of these species was < 1, further suggesting this trap type was less effective than the others we used. Considering only captures in funnel and pitfall traps, 26 species had a significantly different ratio of captures by trap type than the actual ratio of trap types available at the 0.05 level of significance (Table 4). Of these, there were 7 species of frogs with a greater probability of capture in pitfall traps than with funnel traps. Rio Grande leopard frog was the only frog from our study that was more likely to be captured in a funnel trap and was 12 times as likely to be captured in a funnel trap than in a pitfall trap. We had 3 species of lizards that were more likely to be captured in pitfall traps and 2 species of lizards that were more likely to be captured in funnel traps. Eleven of the snake species we detected were more likely to be captured in funnel traps than pitfall traps, with 5 of these being exclusively captured in funnel traps. Flat-headed snake and plains blind snake were more likely to be captured in pitfall traps; the former with 30 of 32 detections from pitfall traps, and the latter with all 20 detections from pitfall traps.

## Discussion

We sampled the herpetofauna at two study sites in southern Texas using a common combination of trap types and drift fencing and validated three critical issues that complicate monitoring efforts. First, despite using standardized techniques considered to be among the best available, estimates for the daily detection probabilities for 12 of the 21 most commonly detected species in our study were < 0.10. Considering we used an intensive effort of 10 days of sampling, our estimates for the cumulative detection probability remained ≤ 0.50 for 10 of the 21 species. The major implications of these low detection rates are that for a modest level of sampling effort, the false absence rate for many species was still > 50%. Additionally, detection rates among species varied substantially, which invalidates key assumptions required to use count data for estimating species richness (Krebs, 1989).

The second issue we confirmed with our study is the significant seasonal variation in detection probability occurring for some species. Our data supported models with seasonal variation for 10 of the 21 most commonly detected species in our study. Seasonal variation in activity patterns are commonly known to occur for many herpetofauna (Ruthven et al., 2002) and should be expected to impact detection probabilities accordingly. Our methods allowed us to generate estimates of the magnitude of variation over our ~ 115-day sampling season. Our results showed that estimates for our 10-day cumulative detection probability varied by > 30% across our sampling season for 6 of the 10 species that we found evidence for seasonal variation in *p*. These results illustrate the likelihood of violating the assumption of constant *p* among sites when the sites are not surveyed simultaneously. Adding to these complicating results, our estimates show that p* for the flat-headed snake was greatest at the beginning of our sampling season, while species including the Gulf Coast toad and Great Plains skink had higher p* towards the middle of our sampling season, and species including Great Plains narrow-mouthed toad and Texas horned lizard had greater p* towards the end of our sampling season. These results illustrate the difficulty in developing monitoring plans to maximize detections for multiple species.

The third issue that our results confirmed is the presence and significant magnitude of variation in *p* that appeared to be caused by rainfall for a range of species. Activity patterns in reptiles and amphibians that in turn affect their detectability are suspected to be closely tied to weather patterns (Jones, 1986; Ruthven et al., 2002). However, we are unaware of any other study that has produced estimates of *p* for various species of herpetofauna over a range of rainfall amounts. While the relationship between *p* and rainfall was positive for 7 species of frogs and toads with a moderate amount of rain, a negative relationship was supported for eastern fence lizard, six-lined racerunner, and Texas glossy snake. Additionally, for the 4 species of frogs and toads with a quadratic relationship with rainfall in the previous 5 days, *p* declined with > 8 cm in the previous 5 days for all 4. Furthermore, our results suggest the impacts of rain on *p* did not immediately dissipate when rain ended; rather, we found measurable impacts 5 days after rain for 6 species and 10 days after rainfall for 2 additional species. These results show despite our attempts to standardize monitoring methods, substantial variation remains, due to factors outside our control.

Our analysis of capture efficiency for each trap type suggests that cover boards were ineffective relative to the other types of traps for all species in our study areas, with one exception. Captures of the Mexican chirping frog were equally likely with cover boards and funnel traps, with 12 and 23 detections from cover boards and funnel traps, respectively. However, capture of this species was still 4 times as likely with pitfall traps as with either of these other methods. Both pitfall and funnel traps were significantly superior for certain species according to our odds ratio calculations. Detections were significantly more likely with funnel traps for 14 species and significantly more likely with pitfall traps for 12 species.

Identification of the most effective trap method for detecting various species of herpetofauna is a common endeavor. Previous studies typically concluded with recommendations involving numerous methods for increasing detections for multiple species and obtaining species richness estimates with reduced bias (Crosswhite et al., 1999; Hutchens & DePerno, 2009; McKnight et al., 2015; Michael et al., 2012; Ryan et al., 2002). Our results also supported the use of multiple methods for increasing detections for a diverse herpetofauna community. For future studies or monitoring focused on multiple species in our study area, we recommend using trap arrays consisting of funnel traps, pitfall traps, and drift fencing similar to what we employed. If maximizing detections of Mexican chirping frog was critical, we suggest also incorporating cover boards. There were a number of species for which we had very few detections that may be due to extremely low occupancy rates. However, many of these species, such as larger snakes, are not efficiently sampled with methods we used and alternative methods, such as larger pitfall traps, would likely improve detection rates (Dodd, 2016; Foster, 2012; Jones, 1986).

## Conclusions

Worldwide declines in herpetofauna, coupled with a paucity of information at the population level, prompts the need for more long-term monitoring of these communities. Monitoring herpetofauna is particularly challenging and guidelines for establishing monitoring programs remain unclear, with some recommending targets of species richness or relative abundance based on indices that require numerous assumptions (Heyer et al., 1994; Smith & Petranka, 2000; JNCC, 2004). Yet others warn against these, favoring what are typically more labor-intensive methods (Dodd & Dorazio, 2004; Hyde & Simons, 2001; Muths et al., 2006). The basic objective of a monitoring program is to collect data that will allow the detection of some difference, typically over time or space. To use an index such as raw counts of captures at the population level, or some estimate of species diversity based on species lists, as the metric for change, detection probabilities must be homogeneous across time or space to draw conclusions with confidence (Williams et al., 2002; Yoccoz et al., 2001). Our results showing low detection probabilities with high variability among species, through the sampling season, and resulting from weather events, is evidence that use of monitoring methods that do not account for these variations will be highly subject to bias. If homogeneous detection probabilities cannot be confirmed, the use of indices for monitoring should only be considered when there is a low likelihood of false absences, which requires high levels of detection probabilities. While we showed that it is possible to achieve an overall detection probability of 0.80 and even 0.95 for most of our 21 most frequently detected species by increasing the duration of sampling at each location, the average time it would take to do so would be 21 and 29 days, respectively. Our results showed that the required duration to reach high levels of *p* may be reduced by adjusting the timing within season for certain species; however, our results also showed that adjusting timing to increase detections for some species will decrease detections for others.

We think it is apparent that the required level of effort and resources to use an index as the primary monitor metric with confidence in the necessary assumptions does not justify the use of the index, particularly with herpetofauna. We agree with the general recommendations for standardizing sampling methods to reduce other sources of variation in *p* (Jones, 1986; Rödel & Ernst, 2004), but further recommend use of analytical methods such as an occupancy framework to identify and account for additional sources of variation that cannot be otherwise controlled. For southern Texas, we recommend monitoring by modeling occupancy similar to what we used in the present study. As it is unlikely to expect less variation in detections within and among species in other systems, we extend this recommendation for monitoring herpetofauna beyond our region. In addition to accounting for imperfect detections and the associated variation therein, use of a modeling framework similar to ours allows additional testing of ecological hypotheses with the potential of increasing our understanding of what drives these populations, as well as the activity levels of individuals (MacKenzie et al., 2006; Muths et al., 2006). Additionally, data collection methods such as we have outlined above, with spatial and temporal replicates, also allows for rigorous estimation of species richness and modeling species interactions. Should these be variables of interest, we recommend using multi-species occupancy models (Dorazio et al., 2006; Devarajan et al., 2020; Tinglet et al., 2020).

**Table 1:** Numbers of captures by year and trap type for all species that we detected on the East Foundation’s San Antonio Viejo and El Sauz ranches in southern Texas, USA, from 2015 and 2016 using drift fence trap arrays. Each array consisted of 3 cover boards, 6 funnel traps, and 4 pitfall traps

		Captures					
Species		By Year			By trap type		
Common	Scientific	2015	2016	Total	Cover board	Funnel	Pitfall
Eastern fence lizard	*Sceloporus undulatus*	27	12	39	0	12	27
Eastern spotted whiptail	*Aspidoscelis gularis*	380	256	636	2	535	99
Four-lined skink	*Plestiodon tetragrammus tetragrammus*	15	13	28	3	12	13
Great Plains skink	*Plestiodon obsoletus*	48	61	109	7	51	51
Little brown skink	*Scincella lateralis*	15	1	16	0	12	4
Ornate tree lizard	*Urosaurus ornatus*	1	0	1	0	1	0
Reticulated collared lizard	*Crotaphytus reticulatus*	1	0	1	0	1	0
Rosebelly lizard	*Sceloporus variabilis*	14	9	23	0	15	8
Six-lined racerunner	*Aspidoscelis sexlineata*	613	474	1087	9	826	252
Slender glass lizard	*Ophisaurus attenuatus*	1	1	2	0	1	1
Texas banded gecko	*Coleonyx brevis*		5	0	5	1	3
Texas horned lizard	*Phrynosoma cornutum*	106	36	142	6	76	60
Texas spiny lizard	*Sceloporus olivaceus*	35	24	59	0	32	27
Black-striped snake	*Coniophanes imperialis*	3	4	7	0	6	1
Cat-eyed snake	*Leptodeira septentrionalis*	1	0	1	0	1	0
Checkered garter snake	*Thamnophis marcianus*	10	12	22	0	20	2
Common kingsnake	*Lampropeltis getula*	1	0	1	0	1	0
Flat-headed snake	*Tantilla gracilis*		24	8	32	0	2
Long-nosed snake	*Rhinocheilus lecontei*	20	13	33	0	32	1
Massasauga	*Sistrurus catenatus*	1	0	1	0	1	0
Mexican Hog-nosed snake	*Heterodon kennerlyi*	1	2	3	0	3	0
Mexican milk snake	*Lampropeltis triangulum annulata*	8	5	13	0	12	1
Mexican racer	*Coluber constrictor oaxaca*	0	1	1	0	1	0
Patch-nosed snake	*Salvadora grahamiae*	16	24	40	0	37	3
Plains Black-headed snake	*Tantilla nigriceps*	0	2	2	0	0	2
Plains rat snake	*Pantherophis emoryi*	8	0	8	0	8	0
Ruthven's whipsnake	*Masticophis schotti ruthveni*	0	2	2	0	2	0
Schott's whipsnake	*Masticophis schotti schotti*	8	4	12	0	12	0
Southwestern rat Snake	*Elaphe guttata meahllmorum*	0	2	2	0	2	0
Texas blind snake	*Leptotyphlops dulcis*	11	9	20	0	0	20
Texas brown snake	*Storeria dekayi texana*	0	1	1	0	1	0
Texas coralsnake	*Micrurus tener*		5	2	7	0	7
Texas glossy snake	*Arizona elegans arenicola*	69	62	131	0	124	7
Texas night snake	*Hypsiglena jani texana*	23	9	32	3	19	10
Texas scarlet snake	*Cemophora lineri*	9	1	10	0	10	0
Western coachwhip	*Masticophis flagellum testaceus*	60	38	98	1	90	7
Western diamondback rattlesnake	*Crotalus atrox*		3	21	24	1	15
Western ground snake	*Sonora semiannulata*	9	4	13	4	3	6
Western ribbon snake	*Thamnophis proximus*	3	5	8	0	8	0
Texas tortoise	*Gopherus berlandieri*	0	1	1	0	0	1
Barred tiger salamander	*Ambystoma mavortium*	2	1	3	0	1	2
Couch's spadefoot	*Scaphiopus couchii*	3	21	24	0	6	18
Great Plains narrow-mouthed toad	*Gastrophryne olivacea*	241	94	335	2	49	284
Gulf Coast toad	*Incilius nebulifer*		83	207	290	1	105
Hurter's spadefoot	*Scaphiopus hurterii*	102	7	109	0	28	81
Mexican chirping frog	*Syrrhophus cystignathoides*	57	40	97	12	23	62
Plains spadefoot	*Spea bombifrons*		14	0	14	0	3
Rio Grande leopard frog	*Lithobates berlandieri*	51	579	630	2	595	33
Sheep frog	*Hypopachus variolosus*	14	35	49	0	19	30
Texas toad	*Anaxyrus speciosus*	17	24	41	1	18	22
Grand Total					2488	2351	4839

**Table 2:** Beta estimates; detection probability (*p*) and associated SE predicted for our median sampling date (T= ordinal day 166) and the mean observed values for daily rainfall (R = 0.24 cm), 5-day accumulated rainfall (5R = 0.98 cm), and 10-day accumulated rainfall (10R = 1.87 cm); and model likelihood (*L*) for the top-supported model for each of the 21 most commonly encountered species on the East Foundation’s San Antonio Viejo and El Sauz ranches in southern Texas, 2015–2016

Species	Beta estimates from model with greatest support^a^									
	Intercept	T	T^2^	R	5R	5R^2^	10R	*p*	SE	*L*^b^
Eastern fence lizard (*Sceloporus undulatus*)	-2.66						-0.16	0.05	0.011	0.93
Eastern spotted whiptail (*Aspidoscelis gularis*)	12.41	-0.15	0.0004					0.22	0.019	1
Great Plains skink (*Plestiodon obsoletus*)	-26.97	0.27	-0.0008					0.11	0.016	1
Keeled earless lizard (*Holbrookia propinqua*)	-0.71							0.33	0.014	1
Six-lined racerunner (*Aspidoscelis sexlineata*)	-1.28	0.0049			-0.098			0.36	0.013	0.69
Texas horned lizard (*Phrynosoma cornutum*)	-4.29	0.012						0.09	0.009	0.25
Texas spiny lizard (*Sceloporus olivaceus*)	-2.65							0.07	0.01	0.24
Flat-headed snake (*Tantilla gracilis*)	-0.75	-0.015						0.04	0.009	1
Long-nosed snake (*Rhinocheilus lecontei*)	-3.32							0.03	0.009	1
Texas glossy snake (*Arizona elegans arenicola*)	-11.78	0.11	-0.0003	-2.066				0.06	0.017	1
Texas night snake (*Hypsiglena jani texana*)	-1.46	-0.012						0.03	0.008	0.95
Western coachwhip (*Masticophis flagellum testaceus*)	-2.87							0.05	0.006	1
Western diamondback rattlesnake (*Crotalus atrox*)	-3.03							0.05	0.012	1
Couch's spadefoot (*Scaphiopus couchii*)	-6.39				1.44	-0.1		0.01	0.004	1
Great Plains narrow-mouthed toad (*Gastrophryne olivacea*)	-10.06	0.05			0.53	-0.038		0.21	0.019	1
Gulf Coast toad (*Incilius nebulifer*)	-101.39	1.17	-0.0034	0.35				0.24	0.021	0.58
Hurter's spadefoot (*Scaphiopus hurterii*)	-1.71				0.53	-0.1		0.22	0.033	0.89
Mexican chirping frog (*Syrrhophus cystignathoides*)	-16.29	0.086			0.71	-0.043		0.18	0.033	1
Rio Grande leopard frog (*Lithobates berlandieri*)	-0.98						0.1	0.31	0.019	1
Sheep frog (*Hypopachus variolosus*)	-2.78				0.21			0.07	0.014	1
Texas toad (*Anaxyrus speciosus*)	-3.18							0.04	0.01	0.5

^a^ empty cells indicate the covariate was not included in the model with greatest support. The covariates of R^2^ and 10R^2^ were also included in candidate models; however, models with these covariates did not receive the greatest support for any species

^b^ Model likelihood of 1 indicates this model received the lowest AIC_c_ score, likelihood < 1 indicates there were competing models within a ΔAIC_c_ of 2 but included a greater number of parameters

**Table 3:** Predicted detection probability for a single day of captures (*p*) and resulting cumulative detection probability (p*) for 4 and 10 days of trapping, as well as the estimated number of days to reach a p* of 0.8 and 0.95 from the East Foundation’s El Sauz and San Antonio Viejo ranches in southern Texas, USA from 2015 and 2016

Species		p*		Days to reach	
	*p*^a^	4 days	10 days	p*=0.8	p*=0.95
Eastern fence lizard (*Sceloporus undulatus*)	0.05	0.19	0.4	31	57
Eastern spotted whiptail (*Aspidoscelis gularis*)	0.22	0.62	0.91	7	12
Great Plains skink (*Plestiodon obsoletus*)	0.11	0.36	0.68	15	26
Keeled earless lizard (*Holbrookia propinqua*)	0.33	0.8	0.98	4	8
Six-lined racerunner (*Aspidoscelis sexlineata*)	0.36	0.84	0.99	4	7
Texas horned lizard (*Phrynosoma cornutum*)	0.09	0.32	0.62	17	30
Texas spiny lizard (*Sceloporus olivaceus*)	0.07	0.24	0.5	24	43
Flat-headed snake (*Tantilla gracilis*)	0.04	0.13	0.3	44	81
Long-nosed snake (*Rhinocheilus lecontei*)	0.03	0.13	0.3	45	82
Texas glossy snake (*Arizona elegans arenicola*)	0.06	0.22	0.46	26	48
Texas night snake (*Hypsiglena jani texana*)	0.03	0.12	0.26	52	95
Western coachwhip (*Masticophis flagellum testaceus*)	0.05	0.2	0.43	29	53
Western diamondback rattlesnake (*Crotalus atrox*)	0.05	0.17	0.38	34	62
Couch's spadefoot (*Scaphiopus couchii*)	0.01	0.02	0.06	>250	>>250
Great Plains narrow-mouthed toad (*Gastrophryne olivacea*)	0.21	0.62	0.91	7	13
Gulf Coast toad (*Incilius nebulifer*)	0.24	0.66	0.93	6	11
Hurter's spadefoot (*Scaphiopus hurterii*)	0.22	0.62	0.91	7	12
Mexican chirping frog (*Syrrhophus cystignathoides*)	0.18	0.55	0.86	8	15
Rio Grande leopard frog (*Lithobates berlandieri*)	0.31	0.78	0.98	5	8
Sheep frog (*Hypopachus variolosus*)	0.07	0.25	0.52	22	40
Texas toad (*Anaxyrus speciosus*)	0.04	0.15	0.33	40	72

^a^Estimates of *p* from models that included rain covariates were generated for our mean observed values of 0.24 cm of rain, 0.98 cm of rain in the previous 5 days, and 1.87 cm of rain in the previous 10 days. Estimates from models that included a time covariate were generated for our mean date of 15 June

**Table 4:** Chi-square (*X*^2^) p-value for testing the hypothesis that captures for each species was independent of trap type, and the relative risk for all three styles used in our drift fence trap arrays on the East Foundation’s San Antonio Viejo and El Sauz ranches in southern Texas, USA from 2015 and 2016. We also tested the hypothesis that captures were independent of trap type considering only captures in funnel traps and pitfall traps and if rejected at the 0.05 level, calculated the relative risk and odds ratio. Each array consisted of 3 cover boards (C-board), 6 funnel traps, and 4 pitfall traps

Species^a^	C-board, funnel traps, and pitfall traps				Funnel traps and pitfall traps				
	*X*^2^	Relative risk			*X*^2^	Relative risk		Odds ratio^b^	
	p-value	C-board	Funnel	Pitfall	p-value	Funnel	Pitfall	Funnel	Pitfall
Eastern fence lizard (*Sceloporus undulatus*)	<0.001	0	0.67	2.25	<0.001	0.51	1.73	0.3	3.38
Eastern spotted whiptail (*Aspidoscelis gularis*)	<0.001	0.01	1.82	0.51	<0.001	1.41	0.39	3.6	0.28
Great Plains skink (*Plestiodon obsoletus*)	<0.001	0.28	1.01	1.52	0.039	0.83	1.25	0.67	1.5
Keeled earless lizard (*Holbrookia propinqua*)	<0.001	0.05	0.38	2.65	<0.001	0.29	2.06	0.14	7.02
Little brown skink (*Scincella lateralis*)	0.034	0	1.63	0.81	0.221				
Rosebelly lizard (*Sceloporus variabilis*)	0.027	0	1.41	1.13	0.61				
Six-lined racerunner (*Aspidoscelis sexlineata*)	<0.001	0.04	1.65	0.75	<0.001	1.28	0.58	2.19	0.46
Texas horned lizard (*Phrynosoma cornutum*)	<0.001	0.18	1.16	1.37	0.327				
Texas spiny lizard (*Sceloporus olivaceus*)	<0.001	0	1.18	1.49	0.366				
Checkered garter snake (*Thamnophis marcianus*)	<0.001	0	1.97	0.3	0.003	1.52	0.23	6.67	0.15
Patch-nosed snake (*Salvadora grahamiae*)	<0.001	0	2	0.24	<0.001	1.54	0.19	8.22	0.12
Flat-headed snake (*Tantilla gracilis*)	<0.001	0	0.14	3.05	<0.001	0.1	2.34	0.04	22.5
Long-nosed snake (*Rhinocheilus lecontei*)	<0.001	0	2.1	0.1	<0.001	1.62	0.08	21.33	0.05
Mexican milk snake (*Lampropeltis triangulum annulata*)	0.004	0	2	0.25	0.017	1.54	0.19	8	0.13
Plains rat snake (*Pantherophis emoryi*)	0.009	0	2.17	0	0.021	1.67	0	11.33	0.09
Schott's whipsnake (*Masticophis schotti schotti*)	0.001	0	2.17	0	0.005	1.67	0	16.67	0.06
Texas blind snake (*Leptotyphlops dulcis*)	<0.001	0	0	3.25	<0.001	0	2.5	0.02	61.5
Texas coralsnake (*Micrurus tener*)	0.017	0	2.17	0	0.031	1.67	0	10	0.1
Texas glossy snake (*Arizona elegans arenicola*)	<0.001	0	2.05	0.17	<0.001	1.58	0.13	11.81	0.08
Texas scarlet snake (*Cemophora lineri*)	0.014	0	2.17	0	0.003	1.67	0	14	0.07
Western coachwhip (*Masticophis flagellum testaceus*)	<0.001	0.04	1.99	0.23	<0.001	1.55	0.18	8.57	0.12
Western ribbon snake (*Thamnophis proximus*)	0.009	0	2.17	0	0.021	1.67	0	11.33	0.09
Couch's spadefoot (*Scaphiopus couchii*)	<0.001	0	0.54	2.44	<0.001	0.42	1.88	0.22	4.5
Great Plains narrow-mouthed toad (*Gastrophryne olivacea*)	<0.001	0.03	0.32	2.76	<0.001	0.25	2.13	0.12	8.69
Gulf Coast toad (*Incilius nebulifer*)	<0.001	0.01	0.78	2.06	<0.001	0.61	1.59	0.38	2.63
Hurter's spadefoot (*Scaphiopus hurterii*)	<0.001	0	0.56	2.42	<0.001	0.43	1.86	0.23	4.34
Mexican chirping frog (*Syrrhophus cystignathoides*)	<0.001	0.54	0.51	2.08	<0.001	0.45	1.82	0.25	4.04
Plains spadefoot (*Spea bombifrons*)	<0.001	0	0.46	2.55	0.003	0.36	1.96	0.18	5.5
Rio Grande leopard frog (*Lithobates berlandieri*)	<0.001	0.01	2.05	0.17	<0.001	1.58	0.13	12.02	0.08
Sheep frog (*Hypopachus variolosus*)	<0.001	0	0.84	1.99	0.002	0.65	1.53	0.42	2.37
Texas toad (*Anaxyrus speciosus*)	0.001	0.11	0.95	1.74	0.052				
Grand Total	<0.001	0.06	1.32	1.23	0.019	1.01	0.95	1.07	0.94

^a^We excluded species with captures that were independent of trap type (*X*^2^ *p* value > 0.05) considering all 3 types

^b^0.5 was added to both capture totals for calculating odds ratios if either was 0

## Data Availability

Data will be made available on reasonable request by the East Foundation, San Antonio, Texas.
